# Incorporation of iloprost in phospholipase-resistant phospholipid scaffold enhances its barrier protective effects on pulmonary endothelium

**DOI:** 10.1038/s41598-018-19197-1

**Published:** 2018-01-17

**Authors:** Olga Oskolkova, Nicolene Sarich, Yufeng Tian, Grzegorz Gawlak, Fanyong Meng, Valery N. Bochkov, Evgeny Berdyshev, Anna A. Birukova, Konstantin G. Birukov

**Affiliations:** 1 Section of Pulmonary and Critical Care Medicine, Department of Medicine, University of Chicago, Chicago, Illinois 60637 USA; 2 Institute of Pharmaceutical Sciences, Department of Pharmaceutical Chemistry, University of Graz, 8010 Graz, Austria; 3 National Jewish Health, Denver, CO 80206 USA; 4 Department of Medicine, University of Maryland School of Medicine, Baltimore, MD 21201 USA; 5 Department of Anesthesiology, University of Maryland School of Medicine, Baltimore, MD 21201 USA

## Abstract

Correction of barrier dysfunction and inflammation in acute lung injury (ALI) represents an important problem. Previous studies demonstrate barrier-protective and anti-inflammatory effects of bioactive lipid prostacyclin and its stable analog iloprost (ILO). We generated a phospholipase resistant synthetic phospholipid with iloprost attached at the *sn*-2 position (ILO-PC) and investigated its biological effects. In comparison to free ILO, ILO-PC caused sustained endothelial cell (EC) barrier enhancement, linked to more prolonged activation of Rap1 and Rac1 GTPases and their cytoskeletal and cell junction effectors: cortactin, PAK1, p120-catenin and VE-cadherin. ILO and ILO-PC equally efficiently suppressed acute, Rho GTPase-dependent EC hyper-permeability caused by thrombin. However, ILO-PC exhibited more sustained barrier-protective and anti-inflammatory effects in the model of chronic EC dysfunction caused by bacterial wall lipopolysacharide (LPS). ILO-PC was also more potent inhibitor of NFκB signaling and lung vascular leak in the murine model of LPS-induced ALI. Treatment with ILO-PC showed more efficient ALI recovery over 3 days after LPS challenge than free ILO. In conclusion, this study describes a novel synthetic phospholipid with barrier-enhancing and anti-inflammatory properties superior to existing prostacyclin analogs, which may be used as a prototype for future development of more efficient treatment for ALI and other vascular leak syndromes.

## Introduction

Pulmonary endothelial cell (EC) barrier precisely regulates transport of fluids and macromolecules across the vessel wall, and EC barrier dysfunction developing during lung infection, injury, and other acute conditions may have devastating consequences such as pulmonary edema and acute respiratory distress syndrome (ARDS). Prostaglandin I_2_, or prostacyclin, is a product of cyclooxygenase-induced conversion of arachidonic acid. PGI2 exerts its biological effects via binding to G-protein coupled receptor IP, which stimulates intracellular production of cyclic AMP^1^. Prostacyclin is a relatively unstable prostaglandin product (*in vivo* half-life 40 sec), and therapeutic applications use its more stable synthetic analogs such as iloprost (ILO) with *in vivo* half-life 20–35 min. Prostacyclin-induced elevation of intracellular cAMP leads to smooth muscle relaxation^2^. This feature of prostacyclin is widely used for therapeutic correction of local and systemic circulation, as well as for relaxation of airway smooth muscle during asthma attacks.

The beneficial effects of prostacyclin extend beyond its cAMP-stimulated hemodynamic and antispastic effects. Prostacyclin and its synthetic analogs, iloprost and beraprost, exhibit direct protective action on the vascular endothelium^3–6^ and significantly reduce lung injury and inflammation induced by high tidal volume mechanical ventilation or bacterial pathogens^7,8^. Barrier-protective and anti-inflammatory properties of prostacyclin and ILO are associated with cAMP-mediated regulation of cytoskeletal dynamics, cell junctions, and inflammatory signaling. In addition to activation of cAMP-activated protein kinase (PKA), increased intracellular cAMP stimulates cAMP-activated guanine exchange factor Epac and its effector GTPase Rap1 which leads to recruitment of Rac-specific guanine nucleotide exchange factors Tiam1 and Vav2 and eventual activation of Rac1 GTPase^4,5,9^. Activation of Rac1 in endothelial cells stimulates peripheral actin polymerization, enforcement of peripheral F-actin rim and increases formation of VE-cadherin-based adherens junctions. These morphological and functional changes lead to enhancement of lung vascular endothelial barrier^5^. In turn, attenuation of agonist-induced EC permeability and inflammation by prostacyclin or ILO is mediated by Rap1/Rac1- and PKA-dependent inhibition of RhoA pathway^5,10^ known to trigger endothelial hyperpermeability and potentiates inflammatory response by enhancing NFkB inflammatory cascade^11^.

Previous studies in the models of acute lung injury and agonist-induced EC barrier dysfunction demonstrated potent and sustained protective effects of oxidized phospholipids bearing iso-prostanoid moiety^12–14^. In this study, we synthesized for the first time phospholipase-resistant synthetic phosphatidyl choline with ILO attached at the *sn*-2 position (ILO-PC). We compared barrier-protective and anti-inflammatory effects of free and phospholipid-bound ILO in human pulmonary EC treated with edemagenic agonist thrombin or bacterial wall lipopolysacharide LPS. Effects of ILO and ILO-PC were further investigated in the *in vivo* model of ALI caused by intratracheal instillation of LPS.

## Results

### Comparison of effects of ILO, ILO-PC, and OxPAPC on EC permeability

**Figure 1:**
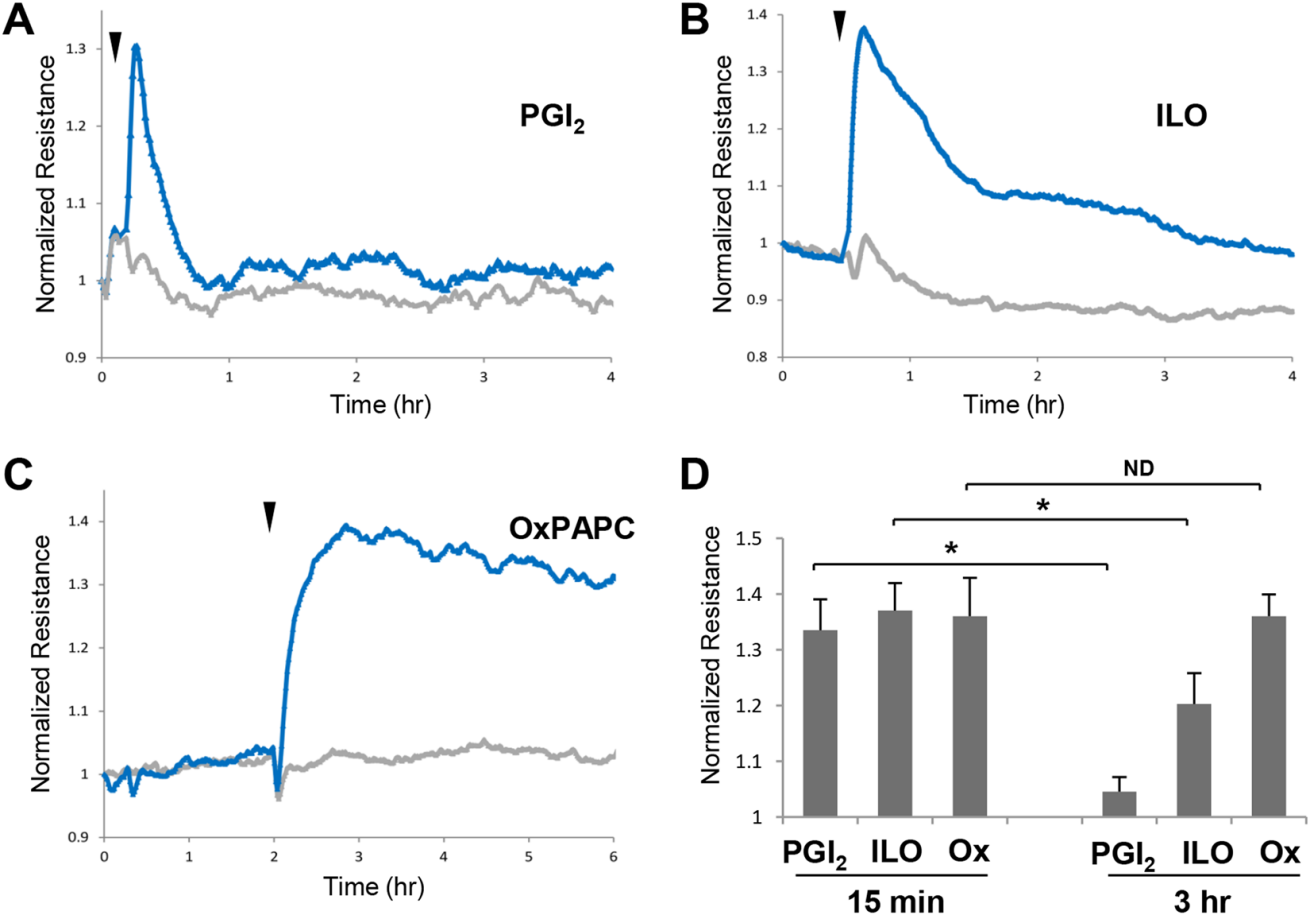
Effects of prostacyclin, iloprost and OxPAPC on pulmonary endothelial cell barrier properties. Pulmonary EC grown on microelectrodes were stimulated with vehicle or: (**A**) 0.5 µM PGI_2_; (**B**) 0.5 µM iloprost (ILO); and (**C**) 15 µg/ml OxPAPC. Changes in transendothelial resistance (TER) were monitored over 4-hr or 6-hr time periods. (**D**) Comparative measurements of TER were performed in EC monolayers stimulated with PGI_2_, ILO and OxPAPC after 15 min and 3 hrs of agonist treatment; n = 5; *p < 0.05. Data are expressed as mean ± SD. Biological replicates indicated by (n). Statistical significance by unpaired Student’s *t*-test.

Previous studies demonstrated barrier-protective and anti-inflammatory effects of PGI_2_ (prostacyclin)^4,10^. However, relatively short (less than one minute) half-life of natural PGI2 after intravenous injection^15^ stimulated search for synthetic PGI2 analogs with more prolonged action, such as beraprost and iloprost (ILO) having a half-life about 30 min^2,16^. Stimulation of human pulmonary EC monolayers with PGI_2_ (Fig. 1A) caused rapid but transient increase in transendothelial electrical resistance (TER) reflecting barrier-enhancing response (Fig. 1A). In turn, ILO treatment caused more sustained TER elevation, which is in line with increased ILO stability (Fig. 1B). Effects of PGI_2_ and ILO were further compared with barrier enhancing effect of oxidized 1-palmitoyl-2-arachidonoyl-*sn*-glycero-3-phosphocholine (OxPAPC) described in our previous studies^12^. In comparison to PGI_2_ and ILO, OxPAPC induced most sustained elevation of TER observed during 4 hrs after treatment (Fig. 1C,D).

### Synthesis and characterization of ILO-PC effects on basal pulmonary EC barrier properties and cytoskeletal remodelling

**Figure 2:**
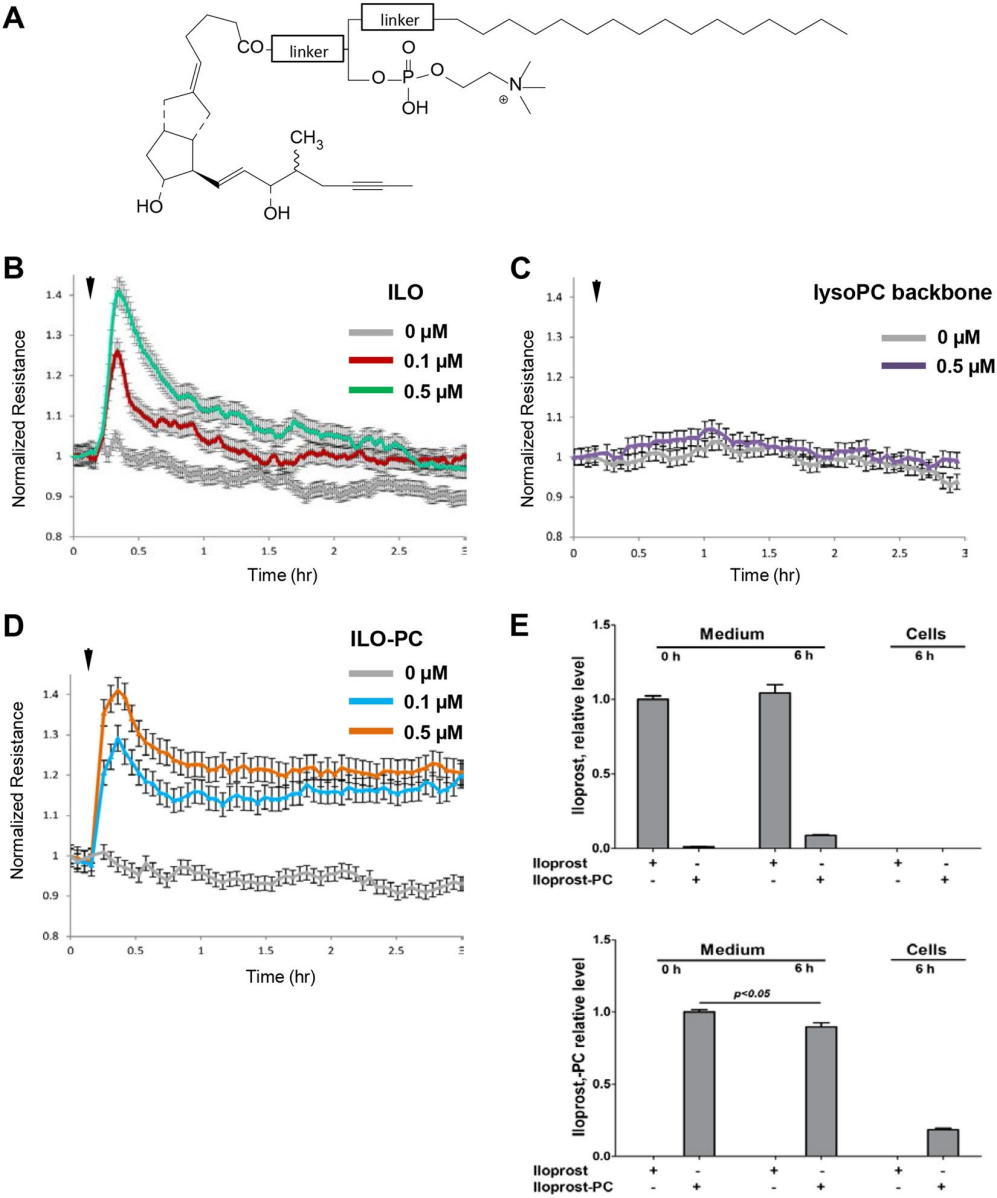
Comparative analysis of time-dependent effects of ILO and ILO-PC on EC barrier. (**A**) Structure of synthetic phospholipid generated by conjugation of iloprost with lysophosphatidylcholine backbone using phospholipase-resistant linker. Agonist-induced EC barrier-enhancing response was evaluated by TER measurements. (**B**) Effect of 0.1 µM and 0.5 µM ILO. (**C**) Effect of 0.1 µM and 0.5 µM lyso-PC backbone used for generation of synthetic ILO-PC. (**D**) Effect of 0.1 µM and 0.5 µM ILO-PC. Shown are pooled TER data expressed as mean +/− SE of four independent measurements. (**E**) Mass Spectrometry analysis of ILO and ILO-PC stability in EC conditioned medium and incorporation in cells. Endothelial cells were treated with 0.4 μM ILO or ILO-PC, and the levels of both compounds were determined in the culture medium at the beginning of incubation and after six hours of treatment. Also, both compounds were determined in cell pellet collected after 6 hrs of agonist treatment. Upper panel: relative levels of ILO in cell culture medium at T = 0 hrs and culture medium and cells at T = 6 hrs. Data are expressed relative to the level of ILO in culture medium at T = 0 hrs (*n* = 4). Lower panel: relative levels of ILO-PC in cell culture medium at T = 0 hrs and culture medium and cells at T = 6 hrs. Data are expressed relative to the level of ILO-PC in culture medium at T = 0 hrs (*n* = 4). Data are expressed as mean ± SD. Biological replicates indicated by (n). Statistical significance by unpaired Student’s *t*-test.

Previous study by our group demonstrated that barrier enhancing effects of OxPAPC were mediated by full length oxygenated products containing isoprostanoid-like moieties^13^. These moieties within natural OxPAPC *in vivo* are prone to cleavage by phospholipases such as lipoprotein-associated PAF-acetyl hydrolase^17^, which may inactivate OxPAPC barrier enhancing activity. On the other hand, presence of oxidatively fragmented molecular species in OxPAPC preparation has adverse effects on EC function^13,17^. In order to circumvent these limitations, we designed a novel synthetic phospholipid. The natural phospholipids contain fatty acids linked via ester bonds at both sn-1 and sn-2 positions to the glycerol backbone. The synthetic ILO-PC molecule has been designed with the ester bond at the sn-1 position and non-ester bond at the sn-2 position linking iloprost moiety rendering its resistance to phospholipase enzymatic activities. Schematic structure of ILO-PC is shown in Fig. 2A. TLC and mass spectrometry analysis confirmed purity of the compound (data not shown). ILO-PC biological activity in comparison to free ILO precursor was further tested in cell culture studies.

Human pulmonary EC monolayers were treated in parallel studies with 0.1 μM and 0.5 μM of ILO or ILO-PC followed by measurements of TER for 3 hrs. EC stimulation with ILO caused dose-dependent, transient increase in TER reflecting EC barrier enhancement, with partial decline by 60 min after treatment (Fig. 2B). EC treatment with lysoPC backbone (0.5 μM) used for synthesis of ILO-PC did not affect EC permeability (Fig. 2C). In contrast to free ILO and lysoPC, cell treatment with synthetic ILO-PC induced sustained TER increase which was observed even after 3 hrs of stimulation (Fig. 2D). Dose-dependent responses to ILO and ILO-PC are also shown in Supplemental Figure 1. Of note, we did not observe further EC TER increase at ILO and ILO-PC concentrations greater than 0.5 µM.

The relative stability of ILO and ILO-PC in conditioned medium was analyzed using mass spectrometry approach described in Methods. Six-hour incubation with endothelial cells with ILO did not significantly decrease its level in conditioned medium (Fig. 2E). At the same time, 6-hr incubation with ILO-PC slightly decreased the levels of ILO-PC (less than 10%) in conditioned medium, but led to ILO-PC accumulation in the cell fraction. In contrast, such accumulation was not observed for free ILO. EC incubation with ILO-PC was also accompanied by release of free ILO (approximately 5%) in the culture medium.

**Figure 3:**
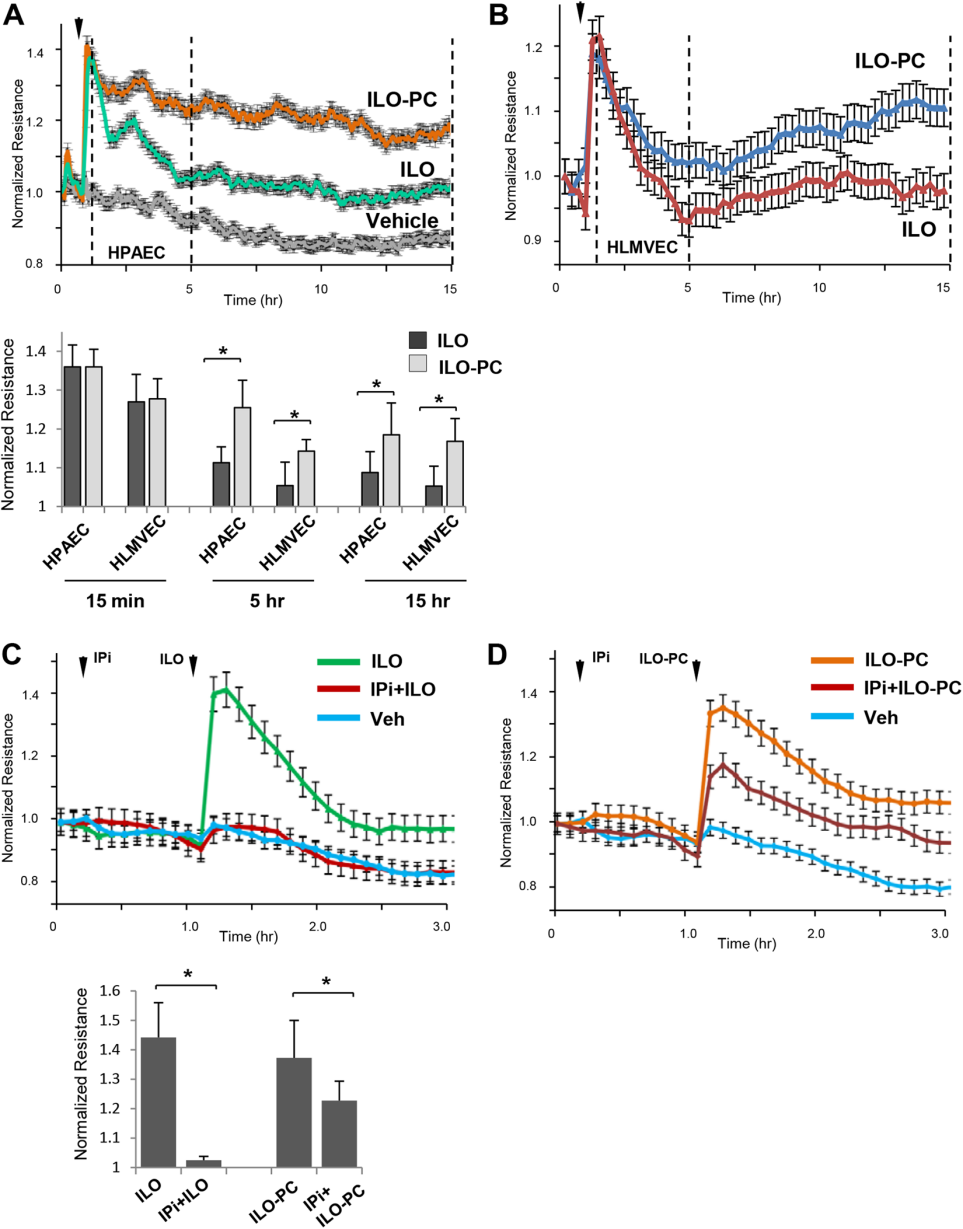
Barrier enhancing effects of ILO and ILO-PC on human lung macrovascular and microvascular EC and the role of prostacyclin receptor. TER measurements of: (**A**) Human pulmonary artery EC (HPAEC) and (**B**) human lung microvascular EC (HLMVEC) stimulated with ILO (0.5 µM) or ILO-PC (0.5 µM). Bar graph depicts quantitative analysis of ILO- and ILO-PC-induced TER changes at the time points after agonist indicated in panels A and B by vertical lines; n = 5; **p < 0.05. (**C**) HPAEC were pretreated with vehicle or prostacyclin receptor small molecule inhibitor (IPi) followed by stimulation with ILO. (**D**) HPAEC were pretreated with vehicle or prostacyclin receptor small molecule inhibitor CAY10441 (IPi, 0.2 µM) followed by stimulation with ILO-PC. Bar graph depicts quantitative analysis of IPi inhibitory effects on ILO- and ILO-PC-induced TER elevation at maximal point. Data are expressed as mean ± SD; n = 5; **p < 0.05. Statistical significance by two-way analysis of variance (ANOVA) and Tukey’s post hoc multiple-comparison test.

Comparative analysis of time-dependent effects of ILO and ILO-PC on pulmonary EC permeability using measurement of TER showed rapid TER increase in response to both, free ILO and ILO-PC (Fig. 3A). In contrast to sustained TER elevation caused by ILO-PC, the TER decline was observed in ILO-treated cells in the first hour. ILO-PC also caused more sustained barrier enhancing response in human lung microvascular EC (Fig. 3B). The pronounced difference in TER levels of ILO- and ILO-PC-stimulated microvascular and macrovascular EC monolayers was observed during the whole 18-hr period of observation (Fig. 3A,B). Bar graph depicts quantitative analysis of ILO- and ILO-PC-induced TER changes at the indicated time points after stimulation. EC response to prostacyclin and its stable analog, ILO, is mediated by G-protein coupled receptor IP. To evaluate IP role in ILO- ILO-PC-induced EC barrier enhancement, we pretreated human lung EC with IP pharmacological inhibitor, CAY10441 (IPi, 0.2 µM) followed by ILO or ILO-PC stimulation. IP inhibitor completely blocked ILO-induced EC barrier enhancing response (Fig. 3C), barrier response to ILO-PC was only attenuated by 50% (Fig. 3D). Bar graph depicts quantitative analysis of IPi suppressing effects on ILO- and ILO-PC-induced TER elevation at maximal point. These results, taken together with results of LC-MS-MS analysis of ILO and ILO-PC stability and differential retention in cell fraction, demonstrate novel functional effects resulting from ILO incorporation into phospholipid molecule.

### Effects of ILO and ILO-PC on Rap1 and Rac1 GTPase signalling

Treatment of pulmonary EC with prostacyclin or ILO elevates intracellular cyclic AMP levels and stimulates cAMP-dependent signaling by protein kinase A (PKA) and cAMP-induced Epac-Rap1-Rac1 GTPase signaling cascade^5^. Rap1 and Rac1 GTPases play a key role in establishment of cell-cell adhesions, enforcement of cortical actin cytoskeleton, and EC barrier enhancement^18^ We therefore investigated the time course of Rac1 and Rap1 activation in ILO- and ILO-PC-stimulated pulmonary EC using pulldown assays.

**Figure 4:**
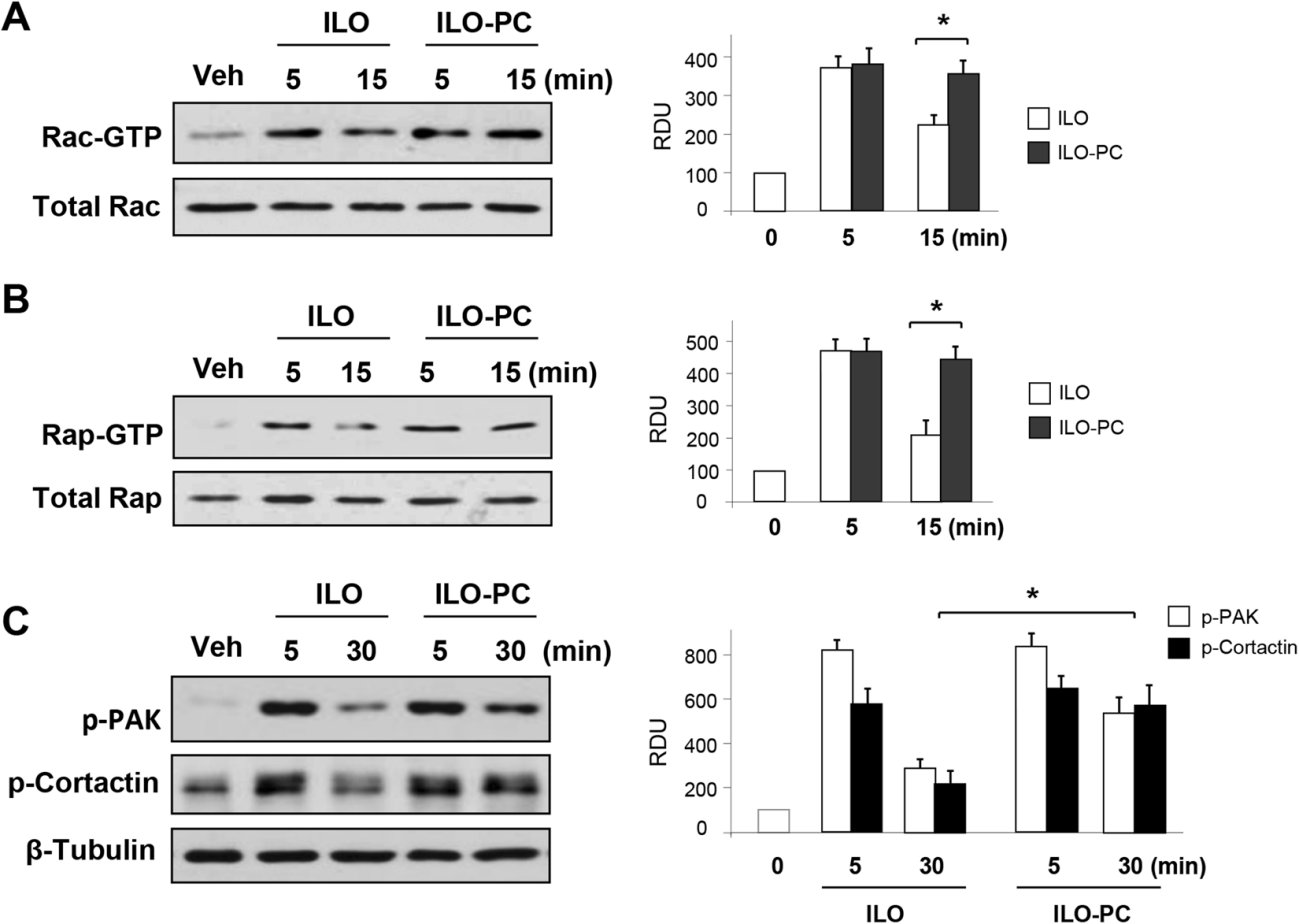
ILO-PC induces more prolonged activation of Rac1 and Rap1 GTPase signaling in pulmonary EC. EC monolayers were stimulated with ILO (0.5 µM) or ILO-PC (0.5 µM) for indicated periods of time. (**A**) Rac-GTP pulldown assay. (**A**) Rap-GTP pulldown assay. (**C**) Phosphorylation of Rac1 targets PAK1 and cortactin was evaluated by western blot with corresponding phospho-specific antibodies. Membrane probing for β-tubulin was used as a normalization control. Bar graphs depict quantitative densitometry analysis of western blot data; n = 3; *p < 0.05. Data are expressed as mean ± SD. Biological replicates indicated by (n). Statistical significance by one-way analysis of variance (ANOVA) and Tukey’s post hoc multiple-comparison test.

Both, ILO and ILO-PC caused prominent rapid activation of Rac1 (Fig. 4A) and Rap1 (Fig. 4B) GTPases, which was observed after 5 min of treatment. Rap1 and Rac1 activation partially declined after 15 min in ILO-treated cells, but remained significantly elevated in ILO-PC-treated ECs. These results indicate more sustained elevation of Rap1 and Rac1 activity caused by ILO-PC.

Rac1-mediated PAK1 activation is manifested by PAK1 autophosphorylation at Thr^428^ ^19^. In addition, tyrosine phosphorylation of another regulator of cortical actin polymerization, cortactin, is also promoted by activated Rac1^20^. Consistent with effects on activation of Rac1, ILO and ILO-PC induced rapid phosphorylation of PAK1 and cortactin, which was transient in the ILO-stimulated EC but remained increased in ILO-PC-stimulated cells (Fig. 4C).

### Effects of ILO and ILO-PC on EC adherens junctions

**Figure 5:**
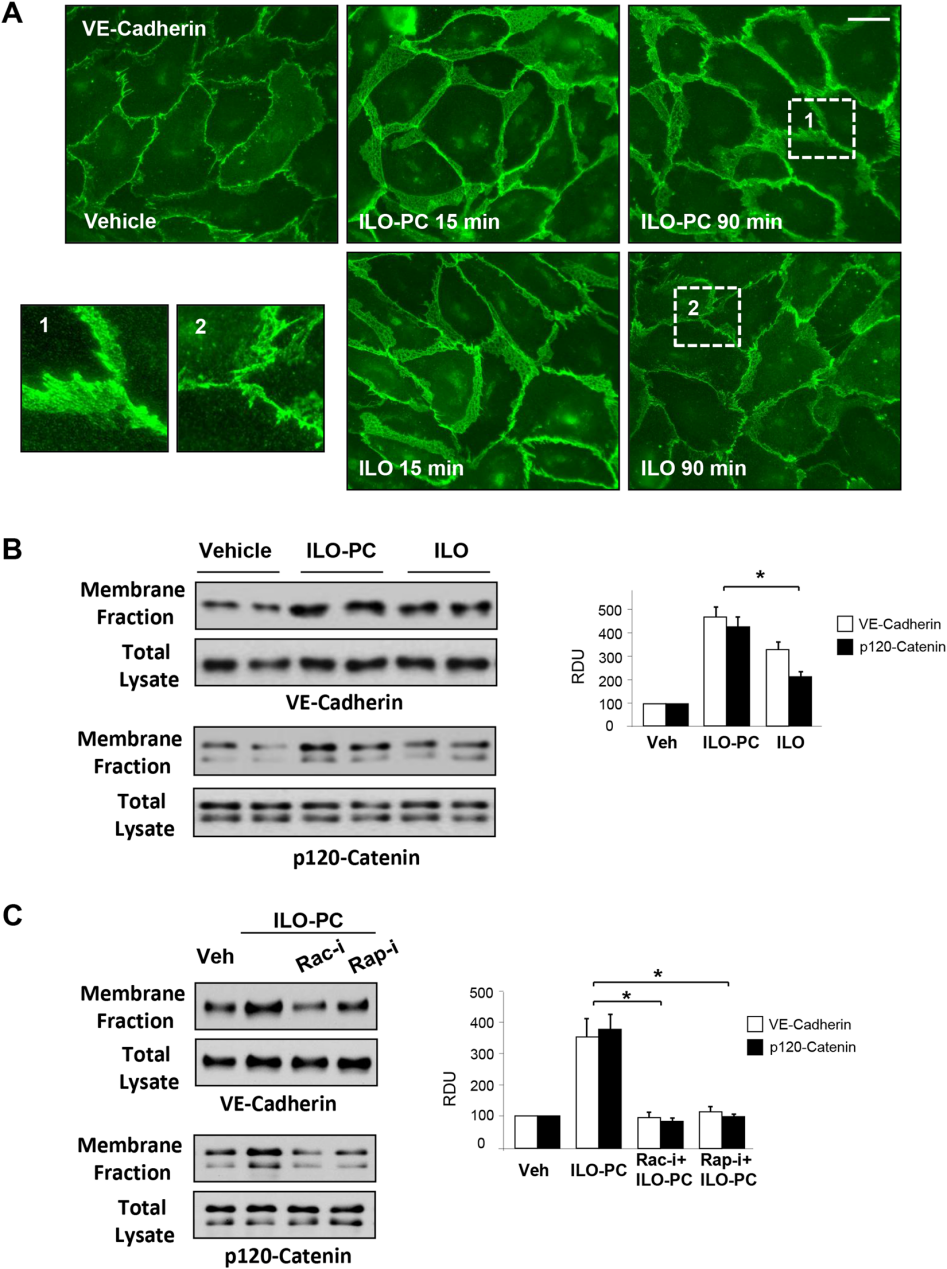
Effects of ILO and ILO-PC on rapid and sustained enhancement of adherens junctions. (**A**) EC monolayers were incubated with 0.5 µM ILO or ILO-PC for 15 or 90 min. Increased areas of adherens junctions in response to agonist treatment were monitored by immunofluorescence staining of transmembrane adherens junction protein, VE-cadherin. Bar = 10 µm. Insets with higher magnification images show details of adherens junction structure. (**B**) Membrane accumulation of adherens junction proteins VE-cadherin and p120-catenin after 90-min stimulation with 0.5 µM ILO or ILO-PC detected by subcellular fractionation assay. (**C**) Membrane accumulation of VE-cadherin and p120-catenin caused by ILO or ILO-PC was analyzed in the presence or absence of Rac1 and Rap1 pharmacological inhibitors (30-min pre-treatment with 100 µM NSC23766 and 10 µM GGTI298, respectively). Probing of total lysates for VE-cadherin and p120-catenin was used as a normalization control. Bar graphs depict quantitative densitometry analysis of western blot data; n = 4; *p < 0.05. Data are expressed as mean ± SD. Biological replicates indicated by (n). Statistical significance by one-way analysis of variance (ANOVA) and Tukey’s post hoc multiple-comparison test.

Remodeling of cell junctions and assembly of adherens junction complexes containing VE-cadherin and p120-, α-, β- γ-catenins is a key mechanism of EC barrier enhancement and barrier restoration^21^. We next characterized the time-dependent remodeling of adherens junctions using biochemical and imaging approaches. Immunofluorescence staining of control, ILO- and ILO-PC-stimulated EC monolayers showed that treatment with either ILO, or ILO-PC dramatically increased areas covered by adherens junctions by 15 min of stimulation, as detected by VE-cadherin-positive immunofluorescence staining (Fig. 5A). However, sustained enhancement of adherens junctions monitored after 90 min of agonist treatment was more pronounced in ILO-PC-stimulated EC monolayers. These data were supported by results of subcellular fractionation, which showed that both, ILO and ILO-PC increased accumulation of EC adherens junction proteins VE-cadherin and p120-catenin in the cell membrane fraction, which indicates increased AJ association. In contrast, this accumulation was less expressed in ILO-treated EC (Fig. 5B). ILO and ILO-PC treatment did not affect total levels of VE-cadherin and p120-catenin in cell lysates in these experiments. Inhibitory analysis using pharmacological inhibitors of Rac1 and Rap1 GTPases showed that, similarly to previously shown effects by free ILO^5,6^, ILO-PC-induced enhancement of cell junctions and accumulation of VE-cadherin and p120-catenin at cell membrane is mediated by Rap1 and Rac1 activities (Fig. 5C).

### Effects of ILO and ILO-PC in cell models of acute and chronic EC barrier dysfunction

**Figure 6:**
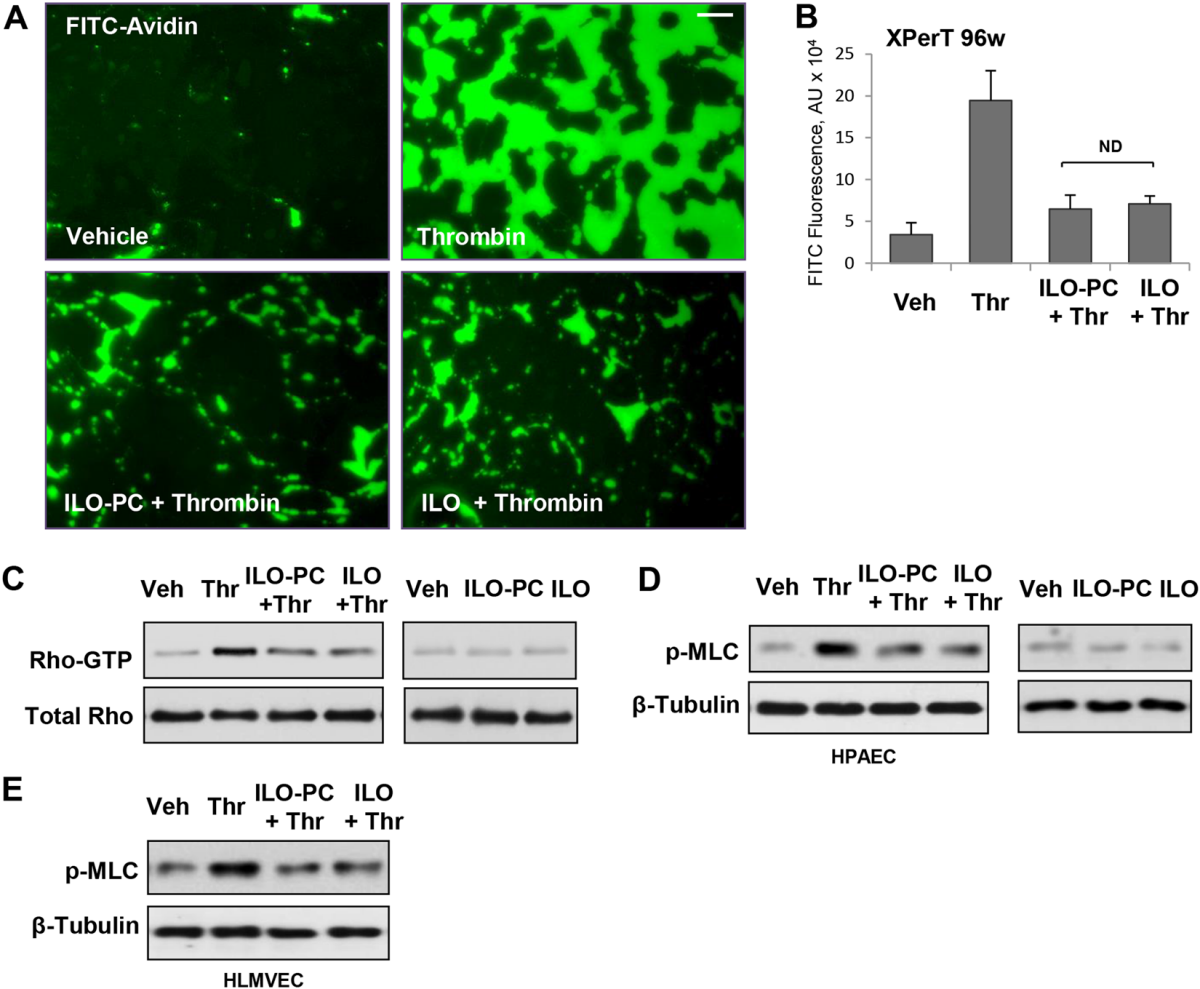
Effects of ILO and ILO-PC on thrombin-induced EC permeability and RhoA pathway activation. EC monolayers were incubated with 0.5 µM ILO or ILO-PC for 15 min prior to thrombin treatment (0.5 U/ml, 5 min). Visualization of FITC-avidin accumulation underneath EC monolayers was performed which reflects an extent of EC barrier dysfunction; bar = 20 µm (**A**). EC permeability for FITC-labeled avidin tracer was assessed by quantitative fluorimetry analysis described in Methods; n = 3; *p < 0.05; ND - no difference (**B**). Pulmonary EC were incubated with 0.5 µM ILO or ILO-PC for 15 min followed by treatment with thrombin or vehicle (C and D left panels). In separate control experiments, the cells were treated with 0.5 µM ILO or ILO-PC alone (C and D right panels). Activation of RhoA GTPase measured by pulldown assays after 5-min thrombin stimulation (**C**). Rho kinase mediated phosphorylation of myosin light chains (MLC) 15 min after thrombin addition was detected by western blot in human pulmonary artery EC (**D**) and human lung microvascular EC (**E**). Probing with antibody to β-tubulin was used as a normalization control. Results are representative of three independent experiments. Data are expressed as mean ± SD. Biological replicates indicated by (n). Statistical significance by one-way analysis of variance (ANOVA) and Tukey’s post hoc multiple-comparison test.

Thrombin-induced permeability in pulmonary vascular EC is a well-known rapid and transient response developing within 5–10 min and followed by complete recovery of EC barrier 40–50 min after thrombin addition^22,23^. Rapid permeability increase caused by thrombin is mediated by RhoA GTPase activation and MLC phosphorylation^24^. Pretreatment with both, ILO and ILO-PC effectively blocked the development of thrombin-induced EC permeability, which was evaluated in FITC-avidin permeability visualization assays (Fig. 6A) and fluorimetry-based quantitative assays in 96-well plates (Fig. 6B). Thrombin-induced increase in EC permeability developed within 5–15 min and was associated with rapid increase in RhoA activity (Fig. 6C) and MLC phosphorylation (Fig. 6D). Both, ILO and ILO-PC blunted thrombin-induced RhoA activation and MLC phosphorylation with comparable efficiency. Alone, ILO or ILO-PC did not activate RhoA signaling (Fig. 6C,D, right panels). Similar suppression of thrombin-induced MLC phospohorylation by free ILO and ILO-PC was observed in human lung microvascular EC (Fig. 6E).

**Figure 7:**
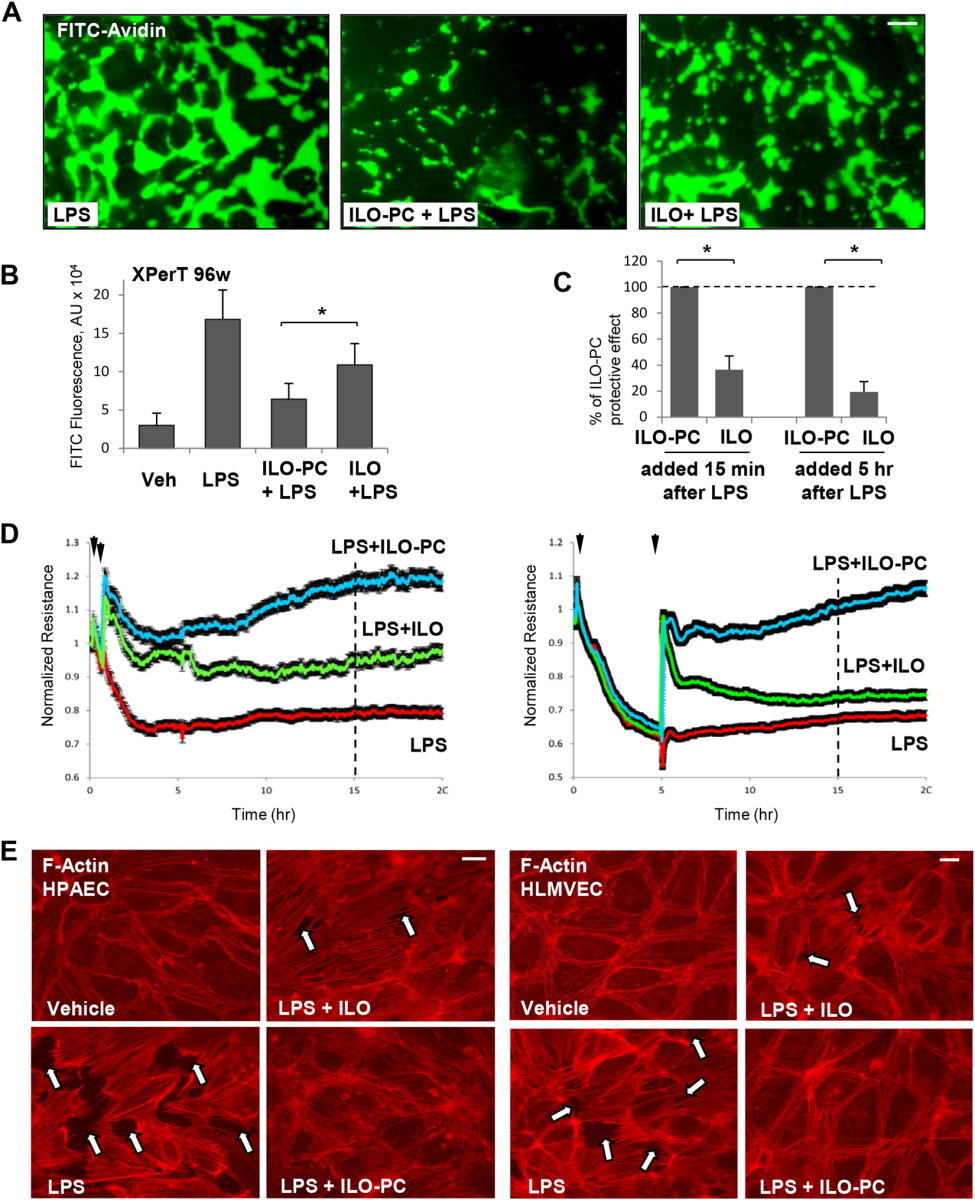
Effects of ILO and ILO-PC on LPS-induced EC barrier dysfunction. Cells were treated with LPS (200 ng/ml) alone or in combination with 0.5 µM ILO or ILO-PC for 6 hrs. (**A**) Visulaization of LPS-induced FITC-avidin binding to substrate underlying EC monolayers reflecting extent of EC barrier dysfunction; bar = 20 µm. (**B**) Analysis of EC monolayer permeability for FITC-avidin was assessed by fluorimetry approach described in Methods; n = 4; *p < 0.05. (**C**) Comparative analysis of effects of ILO- and ILO-PC post-treatment on EC barrier after LPS challenge monitored by TER changes. Agonists were added 15 min or 5 hrs after HPAEC challenge with LPS, and analysis was performed at time points marked by vertical lines in panel D. Protective effect of ILO-PC post-treatment against LPS-induced EC barrier dysfunction at each time point was taken as 100%. Data are expressed as mean ± SD; n = 5; **p < 0.05. Statistical significance by two-way analysis of variance (ANOVA) and Tukey’s post hoc multiple-comparison test. (**D**) Effect of ILO and ILO-PC post-treatment on LPS-induced EC permeability. Pulmonary EC plated on microelectrodes were stimulated with LPS. Vehicle, ILO or ILO-PC was added 15 min (left panel) or 5 hrs after LPS addition (right panel). TER reflecting EC monolayer barrier properties was monitored over 20 hours. (**E**) Attenuation of LPS-induced formation of actin stress fibers and paracellular gaps (marked by arrows) by ILO and ILO-PC in human lung macrovascular (left panels, bar = 10 µm) and microvascular (right panels, bar = 5 µm) EC. F-actin was visualized by cell staining with Texas Red phalloidin. Results are representative of three independent experiments.

EC exposure to LPS causes gradual barrier disruption, which develops within 4–8 hrs and is accompanied by increased expression of inflammatory markers and cytokines^25^. This model was used in the following studies to evaluate effects of ILO and ILO-PC on EC barrier dysfunction in clinically relevant model of LPS-induced inflammation. In contrast to thrombin model of EC permeability, ILO-PC exhibited more pronounced inhibitory effect than free ILO in the model of chronic EC barrier dysfunction caused by LPS. Visualization of FITC-avidin penetration through EC monolayer challenged with LPS for 6 hrs shows better barrier-protective effects of ILO-PC co-treatment, as compared to treatment with free ILO (Fig. 7A). These results were further confirmed by quantitative analysis using 96-well format of this assay (Fig. 7B).

Protective effects of ILO and ILO-PC were also evaluated using TER measurements in the more clinically relevant setting of post-treatment, when agonists were added to EC monolayers 15 min or 5 hrs after LPS challenge. In both experimental settings, ILO-PC exhibited more potent protective effects than free ILO, which were registered after 15 hrs of agonist addition (Fig. 7C). Time-resolved analysis of ILO or ILO-PC effects on LPS-induced EC permeability using TER measurements (Fig. 7D) showed similar levels of rapid EC barrier enhancement by both ILO and ILO-PC in the first 15 mins of treatment. However, ILO-PC exhibited more sustained protection of EC barrier in LPS-stimulated EC than free ILO did. Sustained protective effects of ILO-PC were observed not only after 15 min-post-treatment, but also when ILO-PC was added 5 hrs after LPS challenge (Fig. 7C,D). Morphological analysis of ILO and ILO-PC effects on LPS-induced EC actin cytoskeleton remodeling and barrier disruption was further performed by visualization of F-actin using staining with Texas Red phalloidin. Treatment with ILO-PC for 2 hrs after 5-hr EC exposure to LPS caused complete restoration of EC monolayer integrity and disappearance of intercellular gaps, while protective effect of free ILO post-treatment was weaker, as evidenced by the presence of gaps in treated human lung macrovascular (left panels) and macrovascular (right panels) monolayers (Fig. 7E).

**Figure 8:**
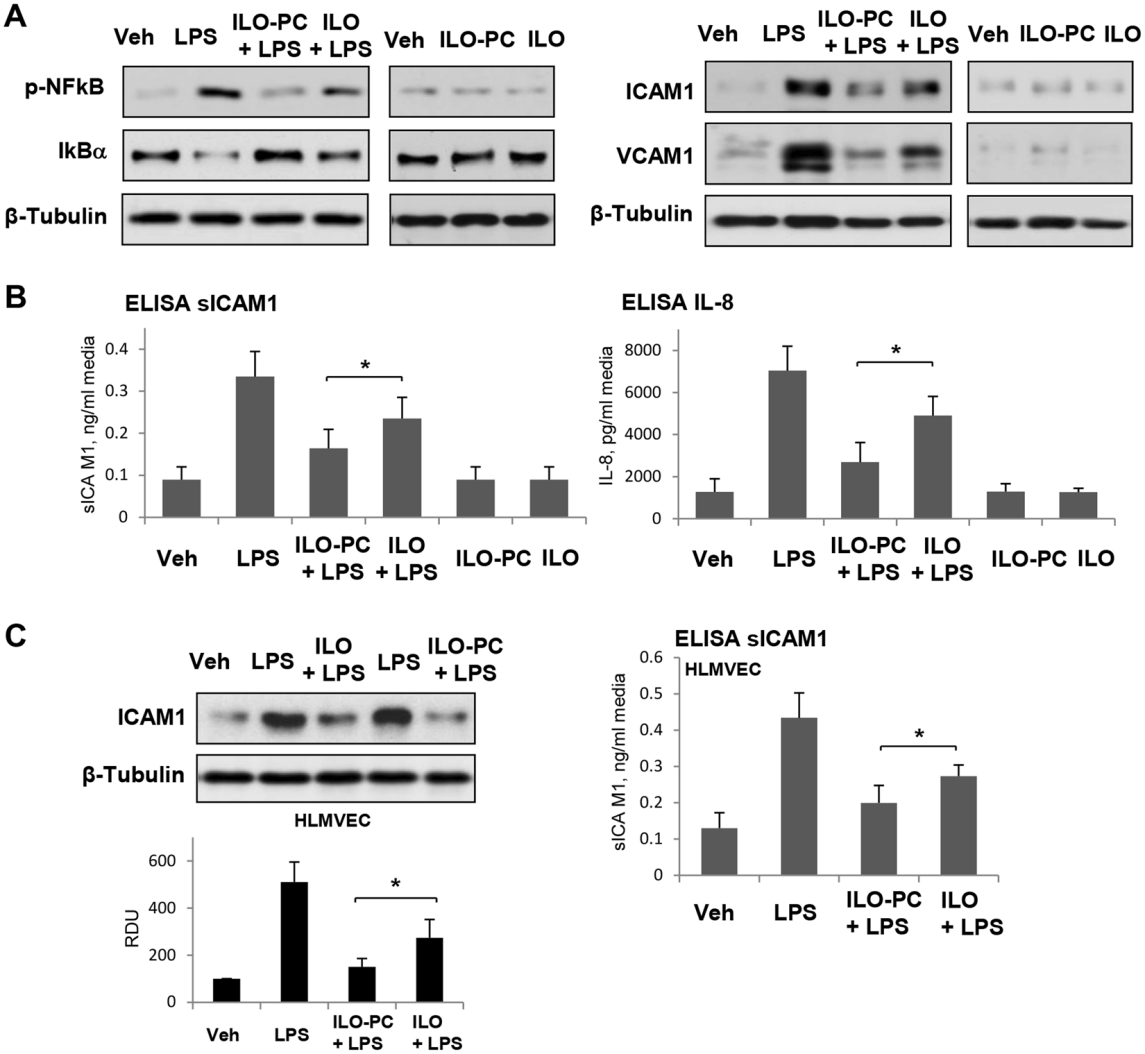
Effects of ILO and ILO-PC on LPS-induced inflammatory activation. Cells were treated with LPS (200 ng/ml) alone or in combination with 0.5 µM ILO or ILO-PC. In separate control experiments, the cells were treated with 0.5 µM ILO or ILO-PC alone. (**A**) IkBα degradation, NFkB phosphorylation (2 hrs after treatment), ICAM1 and VCAM1 expression (6 hrs after treatment) in EC stimulated with LPS, ILO, ILO-PC, or theirs combination was analyzed by western blot. Probing for β-tubulin was used as a normalization control. (**B**) The levels of soluble ICAM1 (sICAM1) and IL-8 in conditioned medium after 6 hrs of HPAEC co-treatment with LPS and ILO or ILO-PC was measured using ELISA assay. (**C**) Expression of cell-bound ICAM1 (left panel) and soluble ICAM1 in conditioned medium after 6 hrs of HLMVEC co-treatment with LPS and ILO or ILO-PC was measured by western blot and ELISA assays; n = 5, *P < 0.05. Data are expressed as mean ± SD. Biological replicates indicated by (n). Statistical significance by one-way analysis of variance (ANOVA) and Tukey’s post hoc multiple-comparison test.

Activation of NFκB-mediated inflammatory signaling cascade is induced by various inflammatory stimuli including LPS and manifested by phosphorylation of NFκB subunit and degradation of inhibitory IκBα subunit. LPS-induced induced phosphorylation of NFκB and degradation of IκBα was attenuated by both, ILO and ILO-PC, although anti-inflammatory effect of ILO-PC was more pronounced (Fig. 8A, left panels).

Activation of vascular endothelium by LPS is known to stimulate NFκB-dependent expression of adhesion molecules ICAM1 and VCAM1 promoting increased neutrophil adhesion and neutrophil transmigration through EC monolayer thus leading to neutrophil recruitment to the inflamed lung tissue. EC treatment with both, ILO and ILO-PC inhibited LPS-induced expression of ICAM1 and VCAM1. However, in agreement with effects on NFκB suppression, ILO-PC was a more potent inhibitor of LPS-induced ICAM1 and VCAM1 expression than free ILO (Fig. 8A, right panels). ILO-PC was also a more potent inhibitor of LPS-activated secretion of soluble ICAM1 and IL-8 by EC exposed to LPS (Fig. 8B). More potent than ILO, anti-inflammatory effects of ILO-PC were also observed in lung microvascular EC (Fig. 8C).

These results clearly demonstrate more sustained and potent protective effects of ILO-PC, as compared to free ILO, against the endothelial barrier dysfunction and inflammatory activation induced by LPS *in vitro*. In the next experiments, we compared protective effects of free ILO and ILO-PC in the murine model of LPS-induced lung injury.

### Effects of ILO and ILO-PC on LPS-induced lung injury

**Figure 9:**
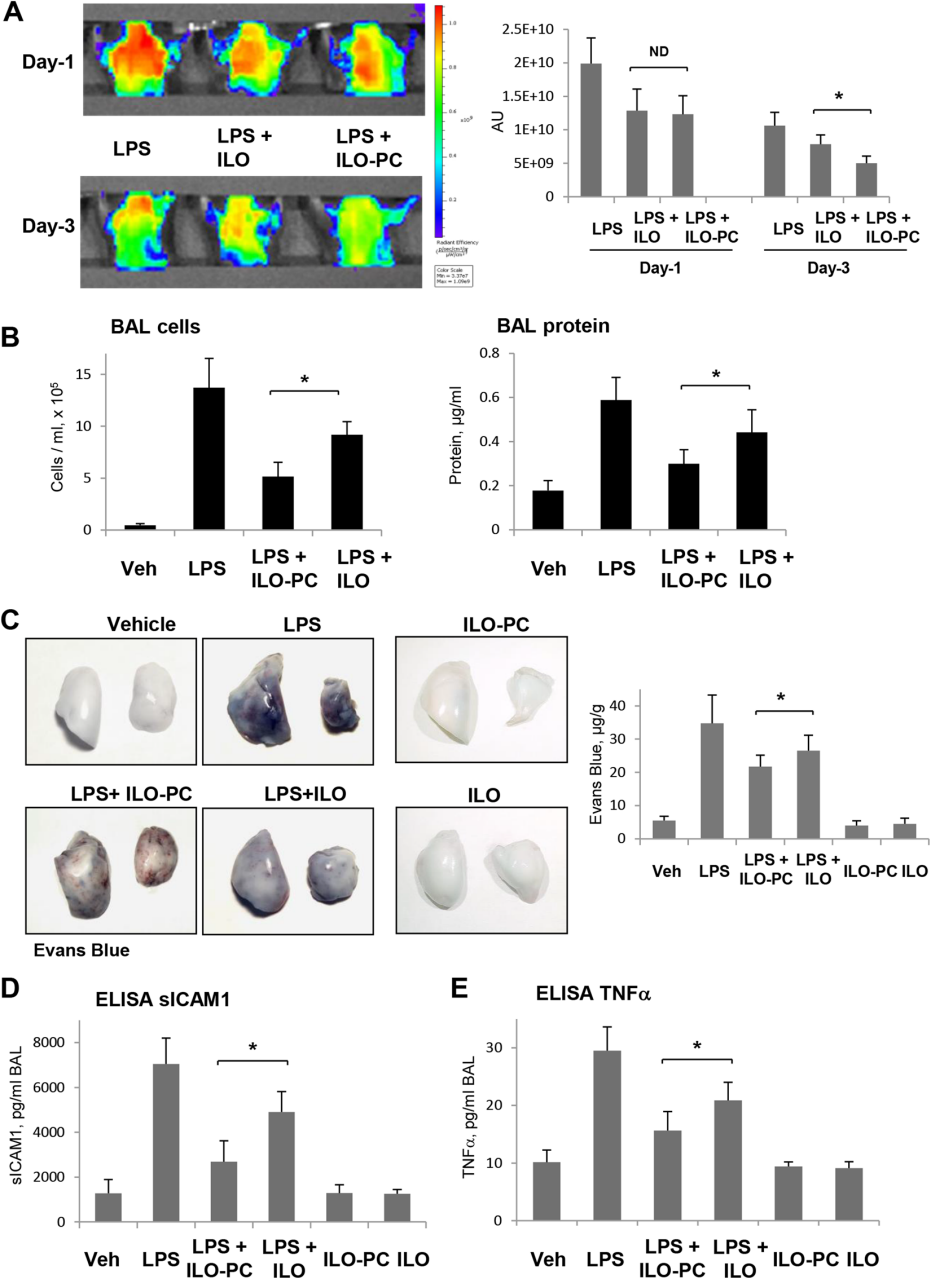
Effects of ILO and ILO-PC on LPS-induced lung injury and barrier dysfunction. (**A**) Live imaging analysis of lung vascular barrier dysfunction after LPS intratracheal injection with and without intravenous administration of ILO or ILO-PC. LPS-induced accumulation of fluorescent Angiosense 680 EX imaging agent in the lungs of same animals was detected by Xenogen IVIS 200 Spectrum imaging system 1 day and 3 days after LPS challenge and presented in arbitrary colors. Bar graph represents quantitative analysis of imaging; n = 4; *p < 0.05. (**B**) Analysis of protein concentration and total cell count was performed in BAL samples obtained from control and experimental groups after LPS challenge; n = 4; *p < 0.05. (**C**) Evans blue dye (30 ml/kg, i/v) was injected 2 hr before termination of the experiment. Photographs depict Evans blue accumulation in the lung tissue illustrating increased lung vascular permeability. Bar graph depicts quantitative analysis of Evans blue labeled albumin extravasation performed by spectrophotometric analysis of Evans blue extracted from the lung tissue samples; n = 4 per condition; *p < 0.05. The levels of soluble ICAM1 (**D**) and TNFα (**E**) were measured in BAL samples using ELISA assay; n = 4; *p < 0.05. Data are expressed as mean ± SD. Biological replicates indicated by (n). Statistical significance by one-way analysis of variance (ANOVA) and Tukey’s post hoc multiple-comparison test.

Mice were treated with LPS followed by concurrent ILO or ILO-PC intravenous injection followed by second injection 5 hrs after LPS challenge. We used non-invasive fluorescence optical imaging approach to evaluate effects of ILO and ILO-PC on LPS-induced lung vascular leak in same animals prospectively, 1 and 3 days after treatment. Angiosense 680 EX tracer was injected intravenously, and tracer accumulation analysis in the lungs reflecting ALI-associated lung vascular leak was performed in anesthetized animals as described in Methods. Angiosense 680 EX accumulation in the lungs was maximal at day-1 after LPS injection and subsided at day-3 (Fig. 9A). Tracer clearance from the injured lungs reflecting lung vascular barrier repair was more evident in the LPS-challenged lungs with ILO-PC treatment as compared to lungs treated with free ILO.

Prominent lung inflammatory response caused by LPS was accompanied by increase in cell count and protein content in bronchoalveolar lavage (BAL) samples (Fig. 9B). These BAL changes still observed at day-3 after LPS treatment were significantly attenuated by ILO and ILO-PC, although ILO-PC displayed more potent protective effect. In consistence with the effects on BAL parameters, treatment with ILO or ILO-PC suppressed Evans Blue dye extravasation in the lung resulting from lung vascular leak. ILO-PC again was more potent in inhibiting LPS-induced vascular leak in comparison to ILO (Fig. 9C).

LPS challenge also significantly increased soluble ICAM1 and TNFα levels detected in BAL samples. In turn, intravenous injection of ILO or ILO-PC significantly attenuated production of TNFα and soluble ICAM1, and ILO-PC demonstrated more pronounced inhibitory effects on these parameters of inflammation (Fig. 9D). Altogether, these data demonstrate more potent protective effects of ILO-PC over free ILO against lung inflammation and barrier dysfunction induced by vasoactive mediators and bacterial pathogens.

## Discussion

This study describes for the first time the biological properties of a phospholipase resistant synthetic phospholipid with incorporated iloprost moiety. Generation of such compound was inspired by previous findings by our group, which demonstrated pronounced and sustained barrier-enhancing and anti-inflammatory effects of isoprostanee-containing products of membrane phospholipid oxidation^13^. These effects of OxPAPC exceeded protective effects of free prostaglandin I_2_ or iloprost (Fig. 1). However, uncertain stability and variable composition of oxidized phospholipid preparations represent a serious obstacle in potential therapeutic application of this group of bioactive lipids. These limitations motivated our efforts in design of an individual phospholipid molecule bearing iloprost, a modified prostacyclin characterized by prolonged action. The results of this study show that incorporation of ILO into synthetic phospholipid did not compromise ILO beneficial effects on vascular endothelial barrier function and its anti-inflammatory properties, but similarly to OxPAPC, led to prolonged EC barrier enhancement and more pronounced anti-inflammatory effect. The lysophospholipid backbone used for ILO-PC synthesis did not enhance EC barrier when added alone at equivalent concentration (Fig. 1), but instead, at higher concentrations it caused modest EC permeability increase (data not shown). These results suggest that ILO conjugation with phospholipid scaffold adds new quantitative and qualitative characteristics to the ILO-PC molecule.

Similarly to free ILO, ILO-PC activated Rac1 and Rap1 GTPases and inhibited thrombin-induced RhoA pathway of EC permeability. In consistence with rapid nature of thrombin-induced EC permeability response associated with acute and transient RhoA activation, ILO-PC did not exhibit superior barrier protective effects over free ILO.

Both ILO and ILO-PC attenuated more sustained LPS-induced barrier dysfunction triggered by inflammatory mechanisms manifested by increased IκBα degradation and NFκB phosphorylation. Such attenuation of inflammatory signaling suppressed LPS-induced ICAM1 and VCAM1 expression in cultured human pulmonary EC and LPS-challenged lungs. These findings strongly suggest that ILO and ILO-PC exhibit their beneficial effects via similar vascular protective and anti-inflammatory mechanisms. However, synthetic ILO-PC revealed several advantages over free ILO or OxPAPC including higher potency and longer effect. First, ILO-PC causes sustained EC barrier enhancement at 50–200 ng/ml concentration range compared to 5–20 µg/ml typical concentration range for OxPAPC. In comparison to free ILO, ILO-PC at the same molar concentration caused more sustained barrier enhancing effect. Second, ILO-PC exhibited more potent barrier-protective and anti-inflammatory effects in the cell and animal models of LPS-induced injury. In addition to analysis of BAL parameters of lung injury, we monitored the time course of lung vascular leak in ILO- and ILO-PC-treated mice with LPS-induced ALI using a non-invasive live imaging approach. The results demonstrated a significant acceleration of lung recovery in mice treated with ILO-PC in comparison to ILO-treated counterparts. These results were consistent with stronger inhibition of LPS-induced Evans blue extravasation reflecting lung vascular leak, accumulation of inflammatory cells, cytokines, and protein levels in BAL samples of ILO-PC-treated mice (Fig. 7).

More potent ILO-PC effects were associated with more sustained activation of Rap1/Rac1 signaling and their cytoskeletal targets resulting in preservation of actin cytoskeletal arrangement and pulmonary EC barrier integrity. This pathway drives barrier-protective and anti-inflammatory effects of prostacyclin and its stable analogs^4,6,8^. Prolonged Rap1/Rac1 activation may also explain better efficiency of ILO-PC in clinically relevant scenario of ILO-PC post-treatment after LPS challenge (Fig. 6).

Sustained EC barrier enhancement and more prolonged activation of Rac1 and Rap1 signaling by ILO-PC is an important finding which warrants further investigation. Interestingly, in contrast to HPAEC, HLMVEC developed induced a bi-phasic barrier enhancement response to ILO-PC, whereby the increase in TER decreased to baseline before increasing again. At present we cannot unequivocally explain the differences in dynamics of barrier enhancement response to ILO-PC by microvascular (HLMVEC) and microvascular (HPAEC) cells, and this aspect deserves further investigation. One may speculate that these differences may reflect: a) donor-to-donor HLMVEC variability in the magnitude of initial spike and following sustained phase; b) phenotypic and functional differences between micro- and microvascular EC reflecting regional heterogeneity of vascular endothelium; or c) differences in gene expression activation between micro-and macrovascular EC developing at a later time point after ILO-PC stimulation.

MS analysis of ILO and ILO-PC in conditioned medium shows similar stability of both compounds. However, we observed retention of ILO-PC, but not free ILO, in EC samples. These findings suggest the increased ILO-PC interaction with cell (in extracellular or intracellular compartment) which may be a factor defining strong long-lasting physiological effect exhibited by ILO-PC. Alternatively, our data showing more sustained barrier enhancing response to ILO-PC and only partial attenuation of this response by IP inhibitor suggest the possibility of less efficient desensitization of IP receptor by ILO-PC, as compared to free ILO, which would lead to more sustained activation of IP receptor signaling. Finally, partial attenuation of ILO-PC response by IP inhibitor, taken together with selective cell uptake of ILO-PC, but not free ILO, indicate a possibility of additional intracellular ILO-PC signaling contributing to EC barrier enhancement and inhibition of LPS-induced EC inflammation. Among factors contributing to more sustained effects of ILO-PC as compared to free ILO may be also activation of additional mechanisms leading to Rap1 and Rac1 activation. As illustration of latter point, oxPAPC, which contains esterified isoprostanes and thus structurally resembles ILO-PC, engages cell membrane-associated pool of GRP78, a chaperone which is increasingly recognized as cell surface signaling receptor^26^ triggering, *inter alia*, Rac1-dependent mechanism of EC barrier enhancement^27^. Thus, precise characterization of unique pharmacodynamics properties and molecular mechanisms underlying long lasting ILO-PC effects is an important follow up of this study and a subject of future research.

In conclusion, we generated and characterized biological properties of a prototype molecule representing a new class of synthetic phospholipids with anti-inflammatory and barrier-protective properties. Our results demonstrate a new way to improve *in vitro* and *in vivo* efficiency of barrier-protective and anti-inflammatory prostaglandin analogs. This novel class of molecules may accelerate recovery in ALI and other inflammatory conditions by improvement of lung vascular barrier function and modulation of innate immune mechanisms and inflammatory signaling cascades.

## Materials and Methods

### Reagents and cell culture

1-Palmitoyl-2-arachidonoyl-sn-glycero-3-phosphocholine (PAPC) was obtained from Avanti Polar Lipids (Alabaster, AL) and oxidized by exposure of dry lipid to air as previously described^28–30^. The extent of oxidation was monitored by positive ion electrospray mass spectrometry (ESI-MS)^30^. PGI_2_, iloprost, and IP receptor inhibitor CAY10441 were obtained from Cayman Chemical (Ann Arbor, MI). Rac1 inhibitor NSC23766 and Rap1 inhibitor GGTI298 were obtained from EMD Millipore. Antibodies to Rac1, Rap1, and p120-catenin were obtained from BD Transduction Laboratories (San Diego, CA); RhoA, VE-cadherin, ICAM1 and VCAM1 antibodies were from Santa Cruz Biotechnology (Santa Cruz, CA); phospho-MLC, phospho-PAK1, phospho-cortactin, phospho-NFκB, and IκBα antibodies were obtained from Cell Signaling (Beverly, MA); antibody to β-tubulin was obtained from Sigma (St. Louis, MO). Texas Red phalloidin and Alexa Flour 488 conjugated secondary antibodies were purchased form Molecular Probes (Eugene, OR). Unless specified, biochemical reagents were obtained from Sigma (St. Louis, MO). Solvents used were of analytical grade. Human pulmonary artery endothelial cells (HPAEC) were obtained from Lonza (East Rutherford, NJ), cultured according to manufacturer’s protocol, and used at passages 5–9.

### ILO-PC synthesis

Amino group of 1-O-hexadecyl-2-amino-2-deoxy-*sn*-glycerol (Bachem, Switzerland) was protected by the Boc group. The protected amino-glycerol was phosphitylated and used for introduction of the choline polar head group as described previously^31^. After the cleavage of the Boc group, the free amino-group was acylated by iloprost succinimide ester. The latter was produced from iloprost (Cayman Chemicals, Ann Arbor, MI) and N-hydroxysuccinimide (Sigma-Aldrich), using EDC (Pierce) as a coupling agent^31^. Purification of the synthesized ILO-PC was performed on an SPE cartridge (SupelClean LC18, Sigma-Aldrich, St. Louis, MO) using a gradient of methanol in water. The purity of the synthesized product was checked by TLC on Kieselgel 60 plates in chloroform-methanol-water (100:50:10, v/v/v) by staining with 10% CuSO4/8.5% H_3_PO_4_ and followed by heating to 120 °C as well as by mass-spectrometry using Sciex 6500 QTRAP triple quadrupole ion trap hybrid mass spectrometer interfaced with Agilent 1290 UHPLC system. Synthetic ILO-PC was dissolved in chloroform and stored at −70 °C. Phospholipid concentration was determined by measurement of organic phosphate as described previously^32^.

### Mass Spectrometry analysis of ILO and ILO-PC in conditioned medium and cell samples

#### Standards and reagents

Methanol, water and chloroform (LC/MS or HPLC grade) were purchased from Thermo Fisher Scientific (Waltham, MA). Lipoxin A4-d5 (LXA4-d5) was purchased from Cayman Chemicals (Ann Arbor, MI), 21:0/22:6-phosphatidylcholine (21:0/22:6-PC) was purchased from Avanti Polar Lipids (Alabaster, AL). The standards were dissolved in methanol and stored at −80 °C.

#### Lipid extraction and sample preparation for LC-MS/MS

Medium and cellular lipids were extracted by modified Bligh and Dyer procedure^33^ with the use of 2% formic acid for phase separation. Internal standards (LXA4-d5 and 21:0/22:6-PC) were added at the beginning of the extraction process.

#### UHPLC-ESI-MS/MS analysis of iloprost and iloprost-PC

The content of iloprost and iloprost-PC were determined by liquid chromatography electrospray ionization tandem mass spectrometry using Sciex 6500QTRAP mass spectrometer coupled with Shimadzu Nexera X2 UHPLC system. Iloprost was detected in negative ions mode as a transition from the m/z 359.1 to the m/z 231.2. Lipoxin A4-d5 was used as the internal standard and detected as a transition from the m/z 356.2 to the m/z 115.0. Chromatography was performed using Ascentis Express RP-Amide 2.7 µm 2.1 × 50 mm column and gradient elution from methanol:water:formic acid (35:65:0.5) to methanol:chloroform:water:formic acid (90:10:0.5:0.5). Iloprost-PC was detected in positive ions mode as a transition from the m/z 823.8 to the m/z 184.1; 21:0/22:6-PC (detected as a transition from the m/z 876.6 to the m/z 184.1) was used as the internal standard for the quantitation of iloprost-PC. Chromatography was performed on the same column using a gradient elution from methanol:water:formic acid (65:35:0.5, 5 mM ammonium formate) to methanol:chloroform:water:formic acid (90:10:0.5:0.5, 5 mM ammonium formate). Standard curves of variable amounts of analytes versus fixed amount of corresponding internal standard were created for the absolute quantitation of iloprost and iloprost-PC.

### Measurements of endothelial monolayer permeability

The cellular barrier properties were analyzed by measurements of transendothelial electrical resistance (TER) across confluent human pulmonary artery endothelial monolayers using an electrical cell-substrate impedance sensing system (Applied Biophysics, Troy, NY) as previously described^34,35^. Endothelial permeability to macromolecules was monitored by permeability visualization assay (XperT)^36^ available from Millipore (Vascular Permeability Imaging Assay, cat. #17–10398). The method is based on high affinity binding of avidin-conjugated FITC-labeled tracer added in cell culture medium during agonist stimulation to the biotinylated ligand in the substrate coating underlying EC monolayers. In permeability visualization experiments, cells grown on biotinylated gelatin-coated glass coverslips were fixed with 3.7% formaldehyde in PBS (10 min, room temperature), and imaging of areas with substrate-bound FITC-avidin was performed.

### GTPase activation assays

Rac, Rap and Rho activation was evaluated in pulldown assays using agarose beads with immobilized PAK1-PBD, Ral GDS-RBD and rhotekin, respectively, as described elsewhere^37^. The levels of activated small GTPases bound to beads and evaluated by Western blot analysis were normalized to total Rac, Rap and Rho levels.

### Immunofluorescence

Endothelial monolayers plated on glass cover slips were subjected to immunofluorescence staining with VE-cadherin antibody as described previously^38^. Texas Red phalloidin was used to visualize F-actin. After immunostaining, slides were analyzed using a Nikon video imaging system (Nikon Instech Co., Tokyo, Japan). Images were processed with Adobe Photoshop 7.0 (Adobe Systems, San Jose, CA) software.

### Differential protein fractionation and immunoblotting

Confluent HPAEC were stimulated with thrombin; cytosolic and membrane fractions were isolated using subcellular protein fractionation kit (Thermo Fisher Scientific, Rockford, IL) according to manufacturer protocol. For analysis of protein phosphorylation profile, cells were stimulated, then lysed, and protein extracts were separated by SDS-PAGE, transferred to polyvinylidene fluoride (PVDF) membrane, and probed with specific antibodies. Equal protein loading was verified by reprobing membranes with antibody to β-tubulin or specific protein of interest. The relative intensities of immunoreactive protein bands (RDU, relative density units) were analyzed and quantified by scanning densitometry using Image Quant software (Molecular Dynamics, Sunnyvale, CA).

### Measurement of cytokines and chemokines

For cytokine measurements in preconditioned medium of human pulmonary EC cultures, supernatants from treated EC were collected and centrifuged to remove debris. IL-8 and soluble ICAM1 (sICAM1) levels were determined by ELISA (R&D Systems, Minneapolis, MN) following manufacturer’s protocol. The concentrations of sICAM1 and TNFα in mouse bronchoalveolar lavage (BAL) fluid samples were measured using mouse-specific ELISA kits (R&D Systems, Minneapolis, MN). Absorbance was read at 450 nm within 30 min in Victor X5 Multilabel Plate Reader (Perkin Elmer, Waltham, MA).

### Animal studies

All animal care and treatment procedures were approved by the University of Chicago Institutional Animal Care and Use Committee. Eight-week old C57Bl/6j mice were purchased from Jackson Laboratories (Bar Harbor, ME). Animals were handled according to the National Institutes of Health Guide for the Care and Use of Laboratory Animals. Bacterial lipopolysaccharide (LPS, 0.63 mg/kg body wt; *Escherichia coli* O55:B5) or sterile water was injected intratracheally in a small volume (20–30 µl) using a 20-gauge catheter (Exelint International, Los Angeles, CA). Iloprost (10 μg/kg), ILO-PC (10 μg/kg) or sterile saline solution was administrated concurrently and 5 hrs after LPS instillation by intravenous injection in the external jugular vein. Animals were sacrificed at day-1 or day-3 by exsanguination under anesthesia. BAL was performed using 1 ml of sterile Hanks balanced salt buffer and measurements of cell count and protein concentration were conducted as previously described^39^. For analysis of LPS-induced lung vascular leak, Evans blue dye (30 ml/kg) was injected into the external jugular vein 2 hrs before termination of the experiment. *In vivo* optical imaging: Mice were injected with 100 µl of 2 nmol Angiosense 680 EX (a vascular fluorescent blood pool imaging agent purchased from PerkinElmer, Boston, MA), intravenously via tail vein. Fluorescence optical imaging was performed in the Integrated Small Animal Imaging Research Resource (iSAIRR) at the University of Chicago using Xenogen IVIS 200 Spectrum (Caliper Life Sciences. Alameda, CA). Mice were exposed to isoflurane anesthesia with O_2_ through the gas anesthesia manifold and placed on the imaging stage. Acquisition and image analysis were performed with Living Image 4.3.1 Software.

### Statistical analysis

Results are expressed as means ± SD of three to five independent experiments. Stimulated samples were compared with controls by unpaired Student’s *t*-test. For multiple-group comparisons, one-way analysis of variance (ANOVA) and Tukey’s post hoc multiple-comparison test were used. P < 0.05 was considered statistically significant.

## Electronic supplementary material

Supplementary Information
