# Supplementary material for: Incorporation of iloprost in phospholipase-resistant phospholipid scaffold enhances its barrier protective effects on pulmonary endothelium
Source: Sci Rep. 2018 Jan 17;8:879. doi: 10.1038/s41598-018-19197-1 (PMC5772615; doi:10.1038/s41598-018-19197-1)
Supplement: Supplementary file 1 — Supplementary Information [file 41598_2018_19197_MOESM1_ESM.doc]

**Supplementary information**

**Incorporation of iloprost in phospholipase-resistant phospholipid scaffold enhances its barrier protective effects on pulmonary endothelium.**

^1,2^Olga Oskolkova, ^1^Nicolene Sarich, ^1^Yufeng Tian, ^1^Grzegorz Gawlak, ^1^Fanyong Meng, ^2^Valery N. Bochkov, ^3^Evgeny Berdyshev, ^4^Anna A. Birukova, and ^5^Konstantin G. Birukov

^1^Section of Pulmonary and Critical Care Medicine, Department of Medicine, University of Chicago, Chicago, Illinois 60637

^2^Institute of Pharmaceutical Sciences, Department of Pharmaceutical Chemistry, University of Graz, 8010 Graz, Austria

^3^ National Jewish Health, Denver, CO 80206

^4^Department of Medicine; and ^5^Department of Anesthesiology, School of Medicine, University of Maryland, Baltimore, MD 21201

**Supplemental Figure S1. Dose-dependent effects of ILO and ILO-PC on EC barrier.** HPAEC monolayers grown on microelectrodes were stimulated with 0.01 µM, 0.05 µM, 0.1 µM and 5 µM of: **A –** ILO; and **B –** ILO-PC. Agonist-induced EC barrier-enhancing response was evaluated by TER measurements. Shown are representative curves of three independent comparative experiments.
